# Magnetic Properties and Morphology Copper-Substituted Barium Hexaferrites from Sol-Gel Auto-Combustion Synthesis

**DOI:** 10.3390/ma14195873

**Published:** 2021-10-07

**Authors:** Abdulmumeen Lohmaah, Komkrich Chokprasombat, Supree Pinitsoontorn, Chitnarong Sirisathitkul

**Affiliations:** 1 Department of Physics, Faculty of Science, Thaksin University, Phatthalung 93210, Thailand; lxabdulmumin@gmail.com; 2 Institute of Nanomaterials Research and Innovation for Energy (IN-RIE), Khon Kaen University, Khon Kaen 40002, Thailand; psupree@kku.ac.th; 3 Department of Physics, Faculty of Science, Walailak University, Nakhon Si Thammarat 80160, Thailand; schitnar@mail.wu.ac.th; 4 Thailand Center of Excellence in Physics, Ministry of Higher Education, Science, Research and Innovation, Bangkok 10400, Thailand

**Keywords:** barium hexaferrite, sol-gel auto-combustion, morphology, magnetization, coercivity

## Abstract

The copper (Cu) substitution in barium hexaferrite (BaFe_12_O_19_) crystals from the sol-gel auto-combustion synthesis is demonstrated as a cost-effective pathway to achieve alterable magnetic properties. Subsequent heat treatments at 450 °C and 1050 °C result in irregularly shaped nanoparticles characterized as the M-type BaFe_12_O_19_ with the secondary phase of hematite (α-Fe_2_O_3_). Despite the mixed phase, the substantial coercivity of 2626 Oe and magnetization as high as 74.8 emu/g are obtained in this undoped ferrite. The copper (Cu) doing strongly affects morphology and magnetic properties of BaFe_12−x_Cu_x_O_19_ (x = 0.1, 0.3, and 0.5). The majority of particles become microrods for x = 0.1 and microplates in the case of x = 0.3 and 0.5. The coercivity and magnetization tend to reduce as Cu^2+^ increasingly substitutes Fe^3+^. From these findings, magnetic properties for various applications in microwave absorbers, recording media, electrodes, and permanent magnets can be tailored by the partial substitution in hexaferrite crystals.

## 1. Introduction

Hexaferrites, composed of iron ions and other divalent metal ions in various atomic ratios, can be classified according to the crystalline structure as M, W, X, Y, Z, and U types. Graphical presentation of these structures can be found in an excellent review [1]. The M-type hexaferrites, especially barium hexaferrite (BaFe_12_O_19_), have been intensively studied and implemented as permanent magnets due to their high intrinsic coercivity (H_c_), large crystalline anisotropy, high chemical stability, and low cost. Properties of barium hexaferrites at high frequencies were implemented in microwave absorbers [2,3]. Their incorporations into polymer composites were investigated to improve forming and mechanical properties [4,5]. There is growing interest in hard/soft magnetic composites with tunable properties, including the combination of barium hexaferrites with magnesium ferrites [6]. Furthermore, potential applications of barium hexaferrites in battery cathodes and magnetic fluids have been proposed [7,8]. Since barium hexaferrite has already been one of the most used magnetic materials with sizeable global market values, its alterable magnetic properties are increasingly being investigated. 

In a magnetoplumbite structure of barium hexaferrites, Fe^3^ ions are located on five different crystallographic sites, and due to the interactions with O^2^ ions, the diverse magnetic properties can be obtained [1]. In addition, the structural substitution by various transition-metal ions modifies magnetic hysteresis loops. Examples are Cu^2+^ [9,10,11,12,13], Co^2+^ [14], Sm^3+^ [15], Ga^3+^ [16], Cr^3+^ [17], and Ce^3+^ [18]. Moreover, the doping of Ce-Co [2], Sm-Co [19], Cu-Zr [20], Co-Zr [21], and La-Mn [22] have been combined in M-type hexaferrites. However, because the magnetic and dielectric properties of ferrites depend on their microstructures and crystal structures depicted in [1,23,24], the different values of coercivity and magnetization have been reported. 

Of particular relevance to this report is the partial substitution in the barium hexaferrite structure by Cu^2+^, which can be either Ba_1−x_Cu_x_Fe_12_O_19_ or BaFe_12−x_Cu_x_O_19_. Asiri et al. synthesized Ba_1−x_Cu_x_Fe_12_O_19_ using the citrate sol-gel combustion method. The coercivity was substantially decreased to 1726 Oe in the case of x = 0.1 but increased from 2121 to 2460 Oe with increasing x from 0.2 to 0.4. By contrast, the saturation magnetization was increased to 54.36 emu/g in the case of x = 0.1 but reduced by higher doping levels [9]. According to the AC susceptibility measurement by Slimani et al. [10], the substitution of Ba^2+^ by Cu^2+^ strongly affected the blocking temperature of Ba_1−x_Cu_x_Fe_12_O_19_. 

For BaFe_12−x_Cu_x_O_19_, the citrate sol-gel combustion method was also used to study the higher x up to 2 [11]. Kumar et al. reported the maximum magnetization and the lowest coercivity in the case of x = 1. The magnetic as well as dielectric properties were correlated with lattice parameters [11]. Alternatively, Rafiq et al. employed the solid-state mixed oxide method. Low coercivities of 932.5 and 262.1 Oe were, respectively, obtained in BaFe_11.9_Cu_0.1_O_19_, and BaFe_11.7_Cu_0.3_O_19_. Interestingly, the coercivity was increased to 1911 Oe with a further increase of Cu to x = 0.5 [12]. 

The research works in [9,10,11,12] demonstrate that Cu doping is a promising route to the commercial production of barium hexaferrites. Variations in sites and levels of partial substitution lead to the coercivity and magnetization suitable for various applications ranging from conventional permanent magnets, recording media, microwave absorbers for novel magnetic fluids, and electrodes. However, the results indicate that the effect of Cu doping on the magnetic properties of the barium hexaferrite needs more investigation. From various methods developed to synthesize and dope ferrites [1,9,10,11,12,13,14,15,16,17,18,19,20,21,22,23,24,25,26], the sol-gel auto-combustion method was selected in this research work to synthesize BaFe_12−x_Cu_x_O_19_ (x = 0, 0.1, 0.3, and 0.5) for its cost-effectiveness [27]. Magnetic properties of BaFe_12−x_Cu_x_O_19_ with Cu doping up to x = 0.5 were compared and correlated to their phase and morphology. The key effects could then be identified and controlled during the synthesis.

## 2. Materials and Methods

Iron(III) nitrate nonahydrate (Fe(NO_3_)_3_⋅9H_2_O), barium nitrate (Ba(NO_3_)_3_), and copper(II) nitrate trihydrate (Cu(NO_3_)_2_⋅3H_2_O) purchased from Sigma-Aldrich and used as received. One mmol of Ba(NO_3_)_3_ and 12 mmol of Fe(NO_3_)_3_⋅9H_2_O were dissolved in 30 mL of deionized water. To prepare BaFe_11.9_Cu_0.1_O_19_ (x = 0.1), 0.1 mmol of Cu(NO_3_)_2_⋅3H_2_O was included. Then, 1.441 g of citric acid was added to the solution and stirred until completely dissolved. Ammonium hydroxide (NH_4_OH) solution (25%) was dropped into the solution to adjust the pH value. After the pH reached 7, the solution was heated at 90 °C for 230 min until a viscous gel was obtained. The gel was then heated at 150 °C for 190 min to allow combustion. The obtained brown powder was ground and subsequently heated in a furnace at 450 °C for 2 h and 1050 °C for 3 h with a heating rate of 4.5 °C/min. Three BaFe_12−x_Cu_x_O_19_ products were obtained with x = 0.1, 0.3, and 0.5, respectively referred to as BaM_0.1, BaM_0.3, and BaM_0.5. A pristine sample (BaM_0.0) was also synthesized by excluding Cu(NO_3_)_2_⋅3H_2_O.

The crystalline structure of the products was examined by X-ray diffractometer (XRD, Philips X′PERT MPD) using 1.5406 Å Cu-kα radiation. The scanning rate was 0.5 degree/min, and the diffraction angle (2θ) was varied between 10° and 90°. The lattice parameters of a hexagonal structure were calculated by the formula [12];

(1) $$\frac{1}{d_{\mathrm{hkl}}^{2}}=\frac{4}{3}\left(\frac{h^{2}+\mathrm{hk}+k^{2}}{a^{2}}\right)+\frac{l^{2}}{c^{2}}$$

where $d_{\mathrm{hkl}}$ is an interplanar spacing as determined by the Bragg formula 2dsinθ=nλ. Whereas h, k, and l are Miller indices, a and c are lattice constants.

The unit cell volume (V_cell_) was then determined from [12]:

(2) $$V_{\mathrm{cell}}=\frac{\sqrt{3}}{2}a^{2}c$$

The crystallite size (D) of BaFe_12−x_Cu_x_O_19_ was calculated using Scherrer’s Formula:

(3) $$D=\frac{K\lambda }{\mathrm{Bcos}\theta }$$

where K is the Scherrer constant, which is 0.89 for hexaferrite, and λ is 1.5406 Å for the Cu-Kα X-ray source. B is full width at half the maximum of the XRD peak, and θ is the XRD peak position (one-half of 2θ). 

A scanning electron microscope (SEM, FEI Quanta 450 FEG, Hillsboro, OR, USA) was used to probe the particle morphology. The as-prepared particles were sputtering-coated with Pd-doped Au before imaging at the accelerating voltage of 10 kV. Energy-dispersive X-ray spectroscopy (EDS, Oxford Instruments, Concord, MA, USA) equipped with the SEM evaluated elemental compositions. Magnetic hysteresis loops were measured by a vibrating sample magnetometer (VSM, VersaLab Quantun Design, San Diego, CA, USA) in sweeping magnetic fields between −30 kOe and +30 kOe at room temperature. From the hysteresis loop, the coercivity was determined from the x-intercept and the magnetic squareness was evaluated from a ratio of the remanent magnetization to the saturation magnetization.

## 3. Results and Discussion

**Figure 1:**
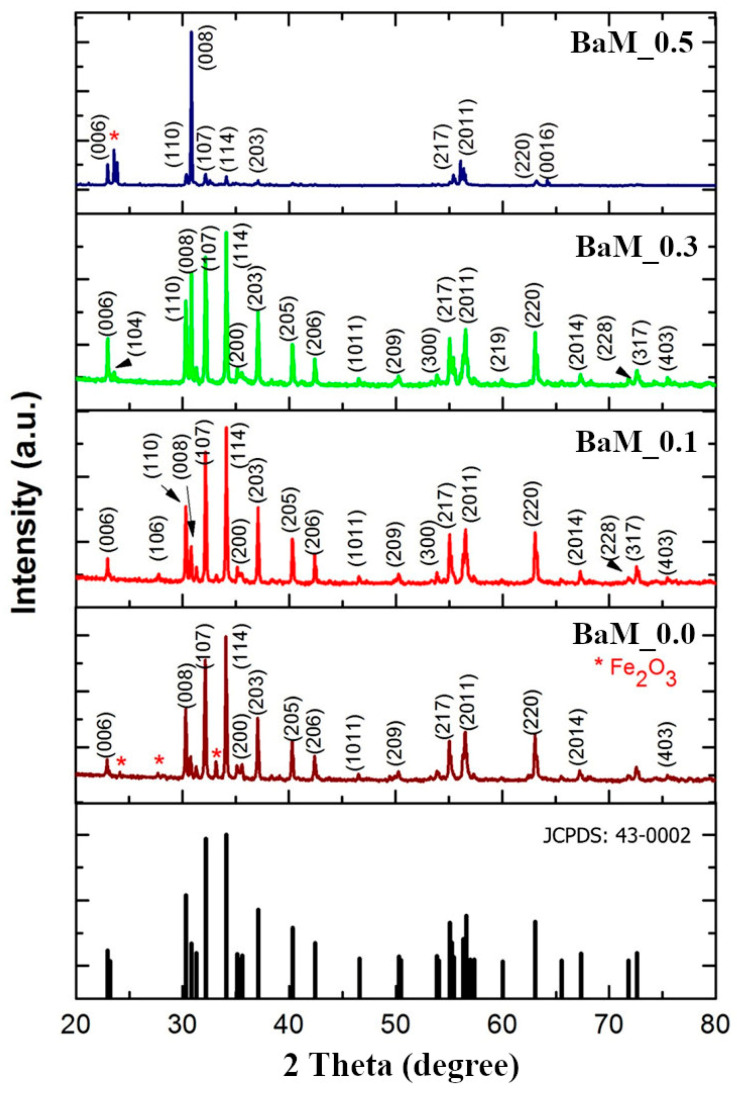
XRD spectra of BaFe_12−x_Cu_x_O_19_ (x = 0, 0.1, 0.3, and 0.5) compared to a standard profile of barium hexaferrite (JCPDS: 43-0002).

All X-ray diffraction patterns in Figure 1 exhibit characteristic peaks of the barium hexaferrite (BaFe_12_O_19_, JCPDS: 43-0002). In the case of pristine M-type hexaferrite (BaM_0.0 sample), the hematite (α-Fe_2_O_3_, JCPDS: 24-0081) phase is also detected in the spectra. This finding is explained in terms of iron oxide formations in the non-substitution case [28]. The substitution by Cu^2+^ tends to promote the sintering process and suppresses iron oxide phases [12]. Only a diffraction peak from the (008) plane of hematite structure is observed in the case of x = 0.5 (BaM_0.5). Other impurity phases often reported in the synthesis of barium hexaferrites such as BaFe_2_O_4_ are not observed.

Parameters from XRD patterns of all samples are listed in Table 1. The c/a ratio and the V_cell_ are not sensitive to the variation in Cu doping from 0.0–0.5. The respective values around 3.94 and 699 Å^3^ are slightly higher than those reported in previous experiments [9,12]. The crystallite sizes calculated from XRD spectra in the case of x = 0.0, 0.1, and 0.3 are 68–72 nm. The increase of Cu to the maximum, x = 0.5, substantially increases the crystallite size to 96 nm. These sizes are more than twice those synthesized by Asiri et al. [9]. Similarly, all particles tend to agglomerate due to their magnetic nature [9]. SEM micrographs clearly show the evolution of BaFe_12−x_Cu_x_O_19_ morphology with increasing substitution by Cu.

**Figure 2:**
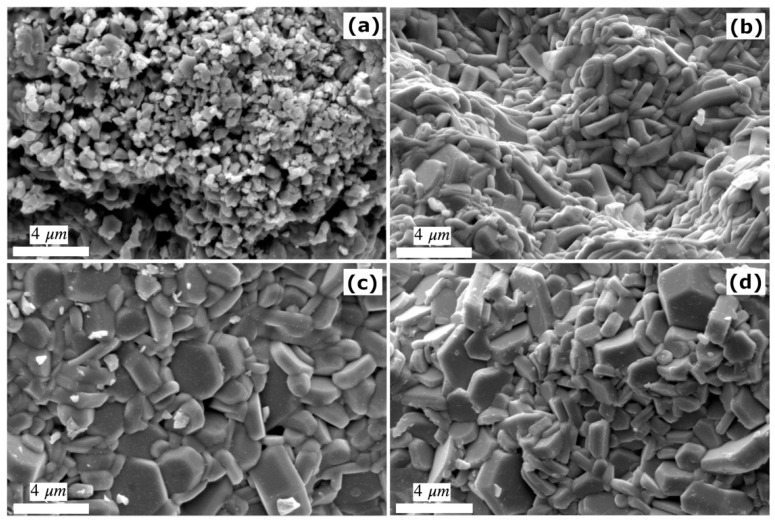
SEM micrographs of BaFe_12−x_Cu_x_O_19_; (**a**) x = 0, (**b**) x = 0.1, (**c**) x = 0.3, and (**d**) x = 0.5.

In the case of x = 0 (BaM_0.0 sample) in Figure 2a, the particles are smallest and irregular in shape. By contrast, the BaM_0.1 sample in Figure 2b mainly contains larger microrods. With increasing Cu doping, the particles in BaM_0.3 and BaM_0.5 samples become plate-like with some hexagonal cross-sections in Figure 2c,d. These particles are densely packed, similar to those reported in [12]. The increase in hexagonal plates and crystallites size at the highest Cu doping is consistent with the suppression of the iron oxide phase depicted by XRD. The trend agrees with a previous report on the effect of Ti substitutions in barium hexaferrite [29]. The effect of substitution on the particle size shown in Figure 2 is more apparent than the normal observation in M-type hexaferrites, which are much more sensitive to calcination [30].

**Figure 3:**
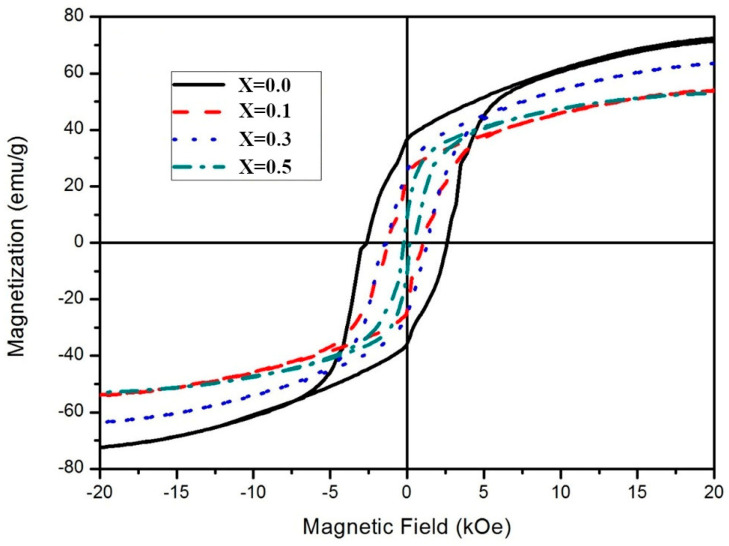
Hysteresis loops of BaFe_12−x_Cu_x_O_19_ (x = 0, 0.1, 0.3, and 0.5) measured by VSM.

Hysteresis loops compare the magnetic properties of BaFe_12−x_Cu_x_O_19_ in Figure 3. The pristine BaM_0.0 sample exhibits the saturation magnetization of 74.8 emu/g and coercivity of 2626 Oe. The saturation magnetization is comparable to the value predicted in single-crystal barium hexaferrite [1] and the values reported by Asiri et al. [9] and Rafiq et al. [12], as listed in Table 2. This high magnetization can be related to the dense structure exemplified in Figure 2. The coercivity in this report is higher than that of BaFe_12−x_Cu_x_O_19_ synthesized by the solid-state mixed oxide route [12] but lower than that of Ba_1−x_Cu_x_Fe_12_O_19_ from the citrate sol-gel combustion [9]. The slight kink in the hysteresis loop when the direction of the magnetic field is reversed corresponds to the presence of the hematite phase in this sample as previously indexed in the XRD spectrum.

According to Table 1, both remanent magnetization and magnetic squareness are substantially reduced by Cu doping. When Cu is doped with x = 0.1, the coercivity is reduced by about a half, as shown in Table 2, which is comparable to the previous report [12]. This decrease is attributed to the reduction of the magnetocrystalline anisotropy due to the 4f_2_ site substitution by Cu^2+^. A further decrease to 343 Oe is observed in the BaM_0.5 sample (x = 0.5). The reduction in coercivity is also related to the morphological change in this report. The enhanced grain size decreases the domain wall pinning sites at the grain boundaries [29]. The difference in hysteresis can be beneficial in electromagnetic wave absorption and magnetic recording applications. 

According to the Ligand field theory, the saturation magnetization increases if Cu^2+^ with d^9^ electrons substitutes Fe^3+^ in the octahedral site [12]. However, the effect of Cu^2+^ doping on the magnetization of barium hexaferrites in this report did not follow the trend predicted by this theory and reported in [9,12], as listed in Table 2. Instead, the saturation magnetization is, respectively, 56.0, 65.9, and 54.4 emu/g for x = 0.1, 0.3, and 0.5. Rafiq et al. and Asiri et al. also observed the reduction in saturation magnetization at the maximum Cu doping of x = 0.5, and Rafiq et al. attributed their results to the substantial increase in c-axis length [9,12]. Such increases are not observed from our Cu doping. The reduction in magnetizations at lower doping levels in this report indicate the effect of Cu^2+^ substitution in different crystallographic sites. The exchange interaction from Fe^3+^-O^2^^−^-Fe^3+^ is weakened as a result of Fe^3+^ oxidation to maintain charge neutrality after doping with nonmagnetic ions and the saturation magnetization is therefore reduced [29]. 

## 4. Conclusions

The structural substitution by Cu^2+^ strongly affected the phase, morphology, and magnetic properties of barium hexaferrite. Using iron(III) nitrate nonahydrate and barium nitrate without doping, the sol-gel auto-combustion synthesis resulted in not only BaFe_12_O_19_ but also the Fe_2_O_3_ phase. Substantial coercivity and saturation magnetization of 2625 Oe and 74.8 emu/g were, respectively obtained. BaFe_12−x_Cu_x_O_19_, where x = 0.1, 0.3, and 0.5, was obtained as the result of copper(II) nitrate trihydrate addition. With increasing x, the morphology changed from irregular nanoparticle clusters to microrods and microplates. The coercivity became 343 Oe, and the saturation magnetization was reduced to 54.4 emu/g by the highest Cu doping (x = 0.5). Future research will be focused on increasing the yield of these barium hexaferrites from each batch of sol-gel auto-combustion. The doping enables the cost-effective production of both bulk magnets and magnetic polymer composites with alterable magnetic properties. Such magnetic materials of different forms and properties will be useful for a variety of applications.

## Figures and Tables

**Table 1:** Comparison of structural parameters from XRD, remanent magnetization, and magnetic squareness from VSM of BaFe_12−x_Cu_x_O_19_ samples.

Sample	c/a Ratio	V_cell_ (Å^3^)	Crystallite Size (nm)	Remanent Magnetization (emu/g)	Magnetic Squareness
BaM_0.0 (x = 0.0)	3.94	699	71	35.8 ± 0.8	0.479
BaM_0.1 (x = 0.1)	3.94	699	68	22.5 ± 1.9	0.402
BaM_0.3 (x = 0.3)	3.94	699	72	25.0 ± 1.1	0.379
BaM_0.5 (x = 0.5)	3.94	697	96	7.2 ± 3.5	0.132

**Table 2:** Comparison of saturation magnetization (Ms) and coercivity (Hc) from the present work to those reported by Asiri et al. [9] and Rafiq et al. [12].

X	Ba_1−x_Cu_x_Fe_12_O_19_ Sol-gel Combustion [9]		BaFe_12−x_Cu_x_O_19_ Solid-State Reaction [12]		BaFe_12−x_Cu_x_O_19_ Sol-gel Combustion [This Work]	
	M_S_ (emu/g)	H_C_ (Oe)	M_S_ (emu/g)	H_C_ (Oe)	M_S_ (emu/g)	H_C_ (Oe)
0.0	48.27	2853	89.0	2263.1	74.8	2626
0.1	54.36	1726	115.0	932.5	56.0	1246
0.2	49.93	2121	-	-	-	-
0.3	53.61	2344	115.1	262.1	65.9	1241
0.4	45.75	2460	-	-	-	-
0.5	40.49	2415	88.5	1911.0	54.4	343

## Data Availability

Data sharing is not applicable.
